# A dynamic programming approach for the alignment of signal peaks in multiple gas chromatography-mass spectrometry experiments

**DOI:** 10.1186/1471-2105-8-419

**Published:** 2007-10-29

**Authors:** Mark D Robinson, David P De Souza, Woon Wai Keen, Eleanor C Saunders, Malcolm J McConville, Terence P Speed, Vladimir A Likić

**Affiliations:** 1 The Walter and Eliza Hall Institute of Medical Research, 1G Royal Parade, Parkville, VIC 3050, Australia; 2 Department of Medical Biology, University of Melbourne, Parkville, VIC 3010, Australia; 3 Department of Biochemistry and Molecular Biology, Bio21 Molecular Science and Biotechnology Institute, University of Melbourne, Royal Parade, Parkville, VIC, 3010, Australia; 4 Bio21 Molecular Science and Biotechnology Institute, University of Melbourne, Royal Parade, Parkville, 3010, Australia; 5 Department of Statistics, University of California, Berkeley, CA 94720-3860, USA

## Abstract

**Background:**

Gas chromatography-mass spectrometry (GC-MS) is a robust platform for the profiling of certain classes of small molecules in biological samples. When multiple samples are profiled, including replicates of the same sample and/or different sample states, one needs to account for retention time drifts between experiments. This can be achieved either by the alignment of chromatographic profiles prior to peak detection, or by matching signal peaks after they have been extracted from chromatogram data matrices. Automated retention time correction is particularly important in non-targeted profiling studies.

**Results:**

A new approach for matching signal peaks based on dynamic programming is presented. The proposed approach relies on both peak retention times and mass spectra. The alignment of more than two peak lists involves three steps: (1) all possible pairs of peak lists are aligned, and similarity of each pair of peak lists is estimated; (2) the guide tree is built based on the similarity between the peak lists; (3) peak lists are progressively aligned starting with the two most similar peak lists, following the guide tree until all peak lists are exhausted. When two or more experiments are performed on different sample states and each consisting of multiple replicates, peak lists within each set of replicate experiments are aligned first (within-state alignment), and subsequently the resulting alignments are aligned themselves (between-state alignment). When more than two sets of replicate experiments are present, the between-state alignment also employs the guide tree. We demonstrate the usefulness of this approach on GC-MS metabolic profiling experiments acquired on wild-type and mutant *Leishmania mexicana *parasites.

**Conclusion:**

We propose a progressive method to match signal peaks across multiple GC-MS experiments based on dynamic programming. A sensitive peak similarity function is proposed to balance peak retention time and peak mass spectra similarities. This approach can produce the optimal alignment between an arbitrary number of peak lists, and models explicitly within-state and between-state peak alignment. The accuracy of the proposed method was close to the accuracy of manually-curated peak matching, which required tens of man-hours for the analyzed data sets. The proposed approach may offer significant advantages for processing of high-throughput metabolomics data, especially when large numbers of experimental replicates and multiple sample states are analyzed.

## Background

Metabolomics refers to an all-inclusive profiling of low-molecular weight metabolites with an implicit aim to interpret results in the context of the organism's genome and its global metabolic network [1-4]. The term metabolic profiling is used to denote either targeted or non-targeted profiling of small molecules in biological samples. A targeted analysis focuses on specific, *a priori *known compounds, and therefore only parts of the chromatogram data matrix generated by the instrument may be considered relevant. In a non-targeted approach all detectable signals are analyzed, and the aim of such analysis is to achieve as wide coverage of metabolites as permitted by a particular experimental technique. A non-targeted metabolic profiling is an essential component of metabolomics, together with bioinformatics approaches for data analysis, interpretation, and integration [1-4]. In recent years metabolic profiling is increasingly being used in studies of microbial metabolism [5], biomarker discovery [6], toxicology [7,8], nutrition [9,10], and integrated systems biology [4,11]. Hyphenated mass spectrometry approaches, in particular gas chromatography-mass spectrometry (GC-MS) [1,12,13] and liquid chromatography-mass spectrometry (LC-MS) [2,14], are often used for metabolic profiling because of their inherent robustness, sensitivity, and large dynamic range.

GC-MS is particularly well suited for studies of low polarity metabolites with high sensitivity and specificity [15]. The processing of GC-MS data is based on the detection of chromatogram signal peaks, a step performed either with proprietary software or with freely available software such as AMDIS [16]. The net result of peak detection is a set of signal peaks that represent components present in the sample. Metabolic profiling experiments are relatively rapid, and typically multiple replicates per sample state are recorded to facilitate robust statistical analyses [1,2,13].

**Figure 1:**
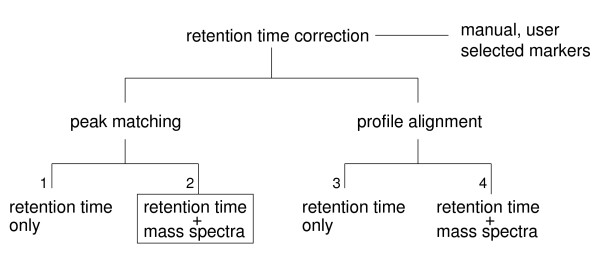
**An overview of methods for retention time correction in hyphenated mass spectrometry profiling experiments**. A technique often used in retention time correction involves spiking internal standards prior to data acquisition, and then linear time correction is applied manually, based on user selected markers [24, 25]. This approach has significant limitations, as discussed in [21]. The automated approaches for retention time correction in hyphenated mass spectrometry are based on two schools of thought: one is to align the entire chromatographic profiles prior to peak detection (profile alignment), and the other is to perform peak detection first, and then match extracted signal peaks across samples to correct for retention time drifts (peak matching). In either approach one can rely on the time domain data only, or include the information from the m/z data domain (mass spectra). Examples of peak matching algorithms that use retention time only (branch 1) include [18, 19, 22]; examples of peak matching algorithms that use both time domain and m/z data (branch 2) include [20, 21, 23]; algorithms for profile alignment that rely on time domain data only (branch 3) were first proposed in 1979 [39], and include [17, 26, 28, 29]; finally, examples of algorithms for profile alignment that use the entire chromatogram data matrices include [27, 30, 37]. The algorithm proposed here is a peak matching approach, and relies on both time domain data and peak mass spectra (branch 2).

**Figure 2:**
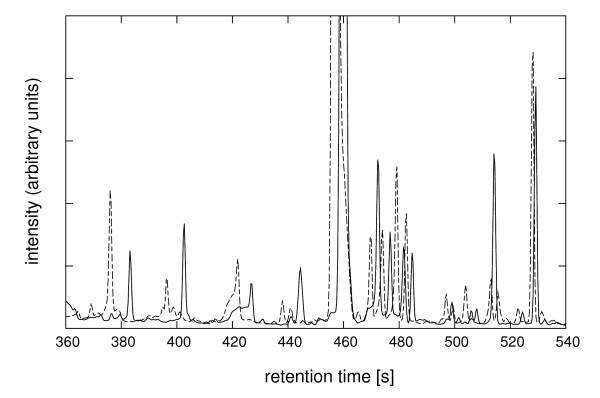
**GC-MS total ion chromatograms showing non-linear time shifts**. A portion of two GC-MS total ion chromatograms (TICs) of polar extracts of *L. mexicana *promastigotes harvested in the mid-log growth phase (solid line) and stationary growth phase (dotted lines). The overlay of raw TICs for the segment between 360 s and 540 s shows non-linear shifts in chromatographic separation often observed in practice.

In order to compare multiple samples, one needs to account for retention time drifts inherent in chromatographic separations [17-23]. The retention time drifts arise because of instrument imperfections (variations in temperature and mobile phase flow rates), column variations (stationary phase saturation and degradation, and stationary phase variations in column-to-column runs), and sample matrix effects (due to variations in sample composition, such as solvent and salts) [17,18]. A review of the literature shows that there is a pressing need for better algorithms for retention time correction in GC-MS small molecule profiling experiments. A summary of approaches used in the past for correcting retention time drifts in hyphenated mass spectrometry experiments is given in Figure 1. An approach often used to correct retention time drifts in practice is a linear correction calculated based on deviations from internal standards [24,25]. This approach has several limitations [21]. For example, addition of internal standards adds new chemicals to the sample, and chromatographic retention time drifts are not linear (Figure 2 and [21]).

The problem of retention time correction is similar in GC-MS and LC-MS small molecule profiling experiments. There are two schools of thought on how to address this: one is to align entire chromatogram profiles prior to peak detection (profile alignment) [26-30], and the other is to perform peak detection first, and then align extracted signal peaks to correct for retention time drifts (peak alignment or peak matching) [18-23]. In either approach one can rely on the time domain data only, or include highly selective information from the m/z data domain (mass spectra).

Both peak matching and profile alignment methods have been used in the context of GC-MS data. Jonsson and co-authors have developed a strategy for rapid comparison of GC-MS metabolic profiles, based on the division of a chromatogram into time windows [28]. To correct the retention time drifts they relied on finding the maximum covariance between the chromatograms [17]. This is a profile alignment method that uses the time domain data only (Figure 1, branch 3). To be effective this method requires that chromatographic profiles to be aligned are similar to one another, as was the case in the analysis of leaf plant extracts where the main purpose of the alignment was to facilitate setting of time domain windows [28]. A different alignment approach was used in the analysis of GC-MS data obtained from the profiling of tomato volatiles, but the algorithm was not described in detail [31].

The peak matching approach is an alternative to the profile alignment that has received a considerable attention in GC-MS [18,19,22]. Johnson et al. matched GC-MS peaks by retention time within a pre-defined time window [18]. Although this approach was shown to be reasonably effective in the GC-MS analysis of diesel fuels, much remains to be desired for applications in biomedical research. For example, it is unclear how to choose the target-chromatogram to which other chromatograms will be aligned; the choice of the cut-off window is arbitrary; and the proposed method of grouping the peaks depends on the order in which the grouping is performed. Duran and co-workers proposed a clustering procedure which groups peaks starting from the minimum retention time [19]. This approach is symmetrical with respect to all experiments and therefore alleviates the need for an arbitrary choice of a target chromatogram. However, the proposed clustering depends on the peak minimum retention time and the arbitrary retention time window [19]. We have attempted to improve on the peak clustering idea by using hierarchical clustering [22]. However, applications of this method have shown that results are rather sensitive to the quality of input data, especially for large retention time drifts. In summary, the body of work on peak matching suggests that accurate peak alignment is unlikely to be achieved by relying on retention time data only.

The most accurate retention time correction is likely to be achieved when complete chromatogram data matrices are utilized. Such methods have been applied to LC-MS [27] and CE-MS [30] metabolic profiling data. The main drawback of any approach that works with entire chromatogram data matrices is the large amount of (uninformative) noise data that must be handled, which dramatically increases computational costs. Our preliminary calculations showed that progressive alignment of chromatogram matrices aimed to avoid the choice of a "target" data set is computationally prohibitive. Furthermore, it is an open question whether the computational costs can be justified by the potential increase in accuracy compared to peak matching methods, when peak matching is based on both retention times and mass spectra.

The most advanced approaches for correction of retention time drifts have been developed on LC-MS and CE-MS data [20,21,27,30]. To our knowledge the peak matching involving mass spectra has not been applied to GC-MS data, except in an approach proposed for a systematic identification of conserved metabolites [23]. This approach aims specifically at identification of conserved metabolites across experiments, and its peak matching is implicitly serving as input for a motif discovery algorithm [23].

Here we present an approach for peak matching that relies on both peak retention times and mass spectra, capable of accurate alignment of peak lists from a large number of replicate GC-MS experiments. We use dynamic programming to arrive at the optimal solution to the global alignment problem. Dynamic programming was previously used for the alignment of chromatogram data matrices [27,30], but not in peak matching. The proposed approach uses a similarity function which balances effectively similarity in peak retention times with the similarity in mass spectra, mimicking the way an experienced human operator performs peak matching. The proposed algorithm is modelled after the heuristic solution for multiple sequence alignment [32,33], and relies on progressive alignment to match an arbitrary number of data sets. We apply this approach to GC-MS metabolic profiling data acquired on wild-type and mutant *Leishmania mexicana *parasites. In the absence of a suitable metric and an accepted benchmark for the peak alignment algorithms, we quantify absolute errors relative to manually derived "true" answer. We show that (*a*) the proposed method performed close to the accuracy of the manually curated alignments by an expert operator; (*b*) the results are not sensitive to the input parameters, suggesting that the proposed method is robust.

## Results

The input for the peak alignment procedure consists of two or more peak lists obtained from hyphenated mass spectrometry experiments (GC-MS data used in this work). These peak lists may be derived from replicate analyses of the same sample (for example, polar extracts from wild-type parasites), or replicate analyses of different samples (for example, polar metabolite extracts of wild-type and mutant parasite lines).

Each experiment is represented by a single and unique peak list. In the most general case, a peak list could be understood as a list of peak objects, where each peak object is characterized with one or more attributes. In the work described here, a peak was characterized with a unique peak ID, the retention time at the peak apex, and the mass spectrum at the peak apex. Henceforth, we assume that a peak list consists of peaks ordered by their retention time. Consider a peak list L_A _that contains peaks p1,p2,...,pnA
 MathType@MTEF@5@5@+=feaafiart1ev1aqatCvAUfKttLearuWrP9MDH5MBPbIqV92AaeXatLxBI9gBaebbnrfifHhDYfgasaacH8akY=wiFfYdH8Gipec8Eeeu0xXdbba9frFj0=OqFfea0dXdd9vqai=hGuQ8kuc9pgc9s8qqaq=dirpe0xb9q8qiLsFr0=vr0=vr0dc8meaabaqaciaacaGaaeqabaqabeGadaaakeaacqWGWbaCdaWgaaWcbaGaeGymaedabeaakiabcYcaSiabdchaWnaaBaaaleaacqaIYaGmaeqaaOGaeiilaWIaeiOla4IaeiOla4IaeiOla4IaeiilaWIaemiCaa3aaSbaaSqaaiabd6gaUnaaBaaameaacqWGbbqqaeqaaaWcbeaaaaa@3B56@. In this case the retention time of the peak p_2 _is greater than that of p_1_, the retention time of the peak p_3 _is greater than that of p_2_, and so on.

### Alignment of two peak lists

Consider two peak lists *L*_*A *_= [p1,p2,...,pnA
 MathType@MTEF@5@5@+=feaafiart1ev1aqatCvAUfKttLearuWrP9MDH5MBPbIqV92AaeXatLxBI9gBaebbnrfifHhDYfgasaacH8akY=wiFfYdH8Gipec8Eeeu0xXdbba9frFj0=OqFfea0dXdd9vqai=hGuQ8kuc9pgc9s8qqaq=dirpe0xb9q8qiLsFr0=vr0=vr0dc8meaabaqaciaacaGaaeqabaqabeGadaaakeaacqWGWbaCdaWgaaWcbaGaeGymaedabeaakiabcYcaSiabdchaWnaaBaaaleaacqaIYaGmaeqaaOGaeiilaWIaeiOla4IaeiOla4IaeiOla4IaeiilaWIaemiCaa3aaSbaaSqaaiabd6gaUnaaBaaameaacqWGbbqqaeqaaaWcbeaaaaa@3B56@] and *L*_*B *_= [q1,q2,...,qnB
 MathType@MTEF@5@5@+=feaafiart1ev1aqatCvAUfKttLearuWrP9MDH5MBPbIqV92AaeXatLxBI9gBaebbnrfifHhDYfgasaacH8akY=wiFfYdH8Gipec8Eeeu0xXdbba9frFj0=OqFfea0dXdd9vqai=hGuQ8kuc9pgc9s8qqaq=dirpe0xb9q8qiLsFr0=vr0=vr0dc8meaabaqaciaacaGaaeqabaqabeGadaaakeaacqWGXbqCdaWgaaWcbaGaeGymaedabeaakiabcYcaSiabdghaXnaaBaaaleaacqaIYaGmaeqaaOGaeiilaWIaeiOla4IaeiOla4IaeiOla4IaeiilaWIaemyCae3aaSbaaSqaaiabd6gaUnaaBaaameaacqWGcbGqaeqaaaWcbeaaaaa@3B5E@] which contain a total number of n_A _and n_B _peaks, respectively. The alignment of the peak lists L_A _and L_B _refers to the establishment of a one-to-one correspondence between the peaks from the two lists, with the possibility that any peak from one list has no matching peak in the other list. The alignment between the peak lists L_A _and L_B _could be represented as a list of peak pairs, where pairing implies peak-to-peak matching. For example,

[(p_1_, q_1_), (p_2_, q_2_), (p_3_, -), (p_4_, q_3_), ...]

where p_1 _is matched with q_1_, p_2 _is matched with q_2_, and so on. The peak p_3 _from the list L_A _does not have a matching peak in the L_B _list. The number of elements in the above list will depend on the optimal alignment, but cannot be less than the larger of A and B, and cannot exceed A+B. For brevity, we refer to the alignment between peak lists L_A _and L_B _as L_A_:L_B_.

It is apparent from the above that the alignment of two peak lists closely resembles the problem of pairwise sequence alignment. The situation for p_3 _in Equation [1] corresponds to matching a sequence letter to a gap. Furthermore, the analogy can be extended even further if one considers that the peak list is an ordered sequence of peaks. The variations in peak retention times arise from various non-linear effects during the separation stage, such as uneven flow of the carrier phase in the GC or LC column. Such perturbations may affect absolute peak retention times and may shift portions of the chromatogram in a non-linear manner [21], but normally do not change the order of peaks in terms of their retention times. The analogy with pairwise sequence alignment implies that dynamic programming could be applied to find the optimal alignment of two peak lists, provided that a suitable scoring scheme can be devised.

### The scoring scheme for peak alignment

In sequence alignment, the cost function for matching two residue letters is obtained from a pre-computed substitution matrix. In the case of peak lists the cost function should reflect similarity between two peaks (henceforth referred to as the peak similarity function). Since the peak mass-spectrum is a key identifier of a particular peak, the peak similarity function should depend heavily on the similarity in the mass spectra. We propose the following peak similarity function P(i,j), which gives the similarity between the peaks i and j:

P(i,j)=S(i,j)⋅exp⁡(−(ti−tj)22D2)
 MathType@MTEF@5@5@+=feaafiart1ev1aaatCvAUfKttLearuWrP9MDH5MBPbIqV92AaeXatLxBI9gBaebbnrfifHhDYfgasaacH8akY=wiFfYdH8Gipec8Eeeu0xXdbba9frFj0=OqFfea0dXdd9vqai=hGuQ8kuc9pgc9s8qqaq=dirpe0xb9q8qiLsFr0=vr0=vr0dc8meaabaqaciaacaGaaeqabaqabeGadaaakeaacqWGqbaucqGGOaakcqWGPbqAcqGGSaalcqWGQbGAcqGGPaqkcqGH9aqpcqWGtbWucqGGOaakcqWGPbqAcqGGSaalcqWGQbGAcqGGPaqkcqGHflY1cyGGLbqzcqGG4baEcqGGWbaCdaqadaqaaiabgkHiTmaalaaabaGaeiikaGIaemiDaq3aaSbaaSqaaiabdMgaPbqabaGccqGHsislcqWG0baDdaWgaaWcbaGaemOAaOgabeaakiabcMcaPmaaCaaaleqabaGaeGOmaidaaaGcbaGaeGOmaiJaemiraq0aaWbaaSqabeaacqaIYaGmaaaaaaGccaGLOaGaayzkaaaaaa@509C@

In the above equation S(i,j) is the similarity between the mass spectra of the peaks i and j, t_i _and t_j _are retention times of peaks i and j, and D is the retention time tolerance parameter which determines the importance of retention times to the overall peak similarity score. The function S(i,j) can be any function that returns a measure of the similarity in m/z ions detected in mass spectra of the peaks i and j. In our test implementation, S(i,j) was calculated as the cosine of the angle between the two mass spectra vectors (i.e. the normalized dot product). This resulted in values between 0 and 1: when the two mass spectra are identical S(i,j) = 1, and when they are completely dissimilar S(i,j) = 0.

The second term in the equation [2] modulates the similarity in mass spectra. When two peaks have identical retention times (t_i _= t_j_) this term equals one, and the peak similarity reduces to the mass spectra similarity (P(i,j) = S(i,j)). When the retention times of the two peaks differ, then exp[-(t_i _- t_j_)^2^/2D^2^] < 1, and P(i,j) < S(i,j). The greater the retention time difference the peak similarity P(i,j) is more reduced relative to the mass spectra similarity S(i,j). The extent of this reduction depends on the relationship between the difference (t_i _- t_j_) and the parameter D. When (t_i _- t_j_) >> D then exp[-(t_i _- t_j_)^2^/2D^2^] will be close to zero and peak similarity will be close to zero (P(i,j) = 0), even if mass spectra are identical (S(i,j) = 1).

The peak similarity function gives the "cost" of matching any two peaks. In addition to this, the dynamic programming algorithm requires a gap penalty to be defined. In sequence alignment gaps are treated with the affine gap function, designed to penalize gaps and to favor the extension of existing gaps over the creation of new gaps. In peak alignment favoring gap extensions is not physically justified, therefore, we set the gap penalty to a fixed number (G). With the above definition of P(i,j) (equation [2]) the meaningful range of G is between 0 and 1. A low value of G would favor the insertion of gaps even when peaks are originating from the same chemical compound; a high value of G would favour the alignment of peaks that are actually different.

With the above definitions for the peak similarity function and the choice of gap penalty it is possible to deploy dynamic programming to find the global solution to the problem of optimal alignment of two peak lists. A standard dynamic programming procedure involves the following steps: (1) initialization of a two dimensional score matrix whose rows are indexed with the peaks of one peak list and columns are indexed with the peaks of the other peak list; (2) filling the cells of the score matrix based on the peak similarity function and possibly the gap penalty; (3) the best alignment between two peak lists is deduced from the traceback on the score matrix. We note that the pairwise alignment of two peak lists is analogous to the problem of global sequence alignment [34].

**Figure 3:**
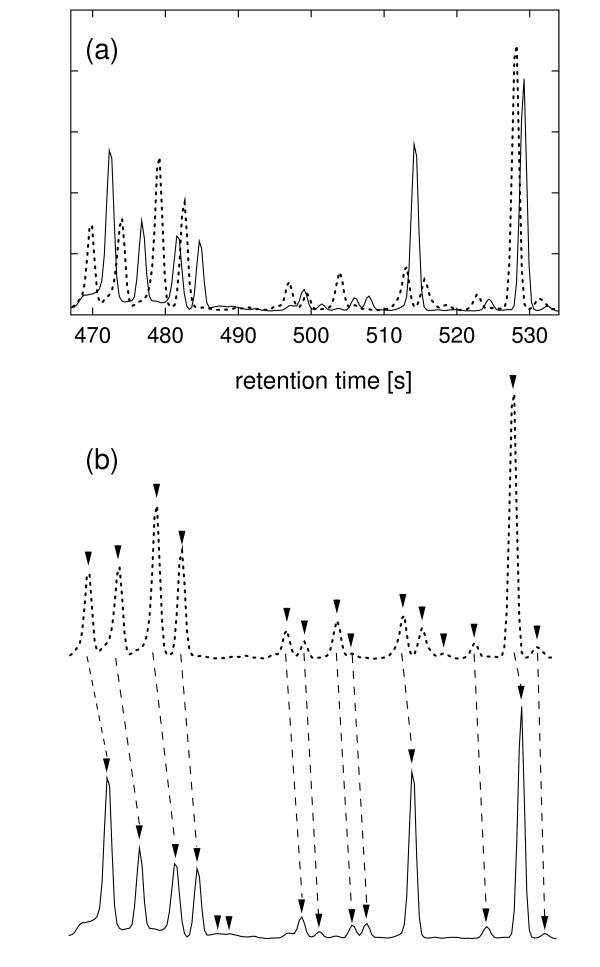
**An example total ion chromatogram alignment**. Panel a. Detail of the segment between 467 s and 534 s of the TICs showed in Figure 1. Fourteen signal peaks were detected in each segment. Panel b. The peak matching after dynamic programming peak alignment (see also Figure 4).

**Figure 4:**
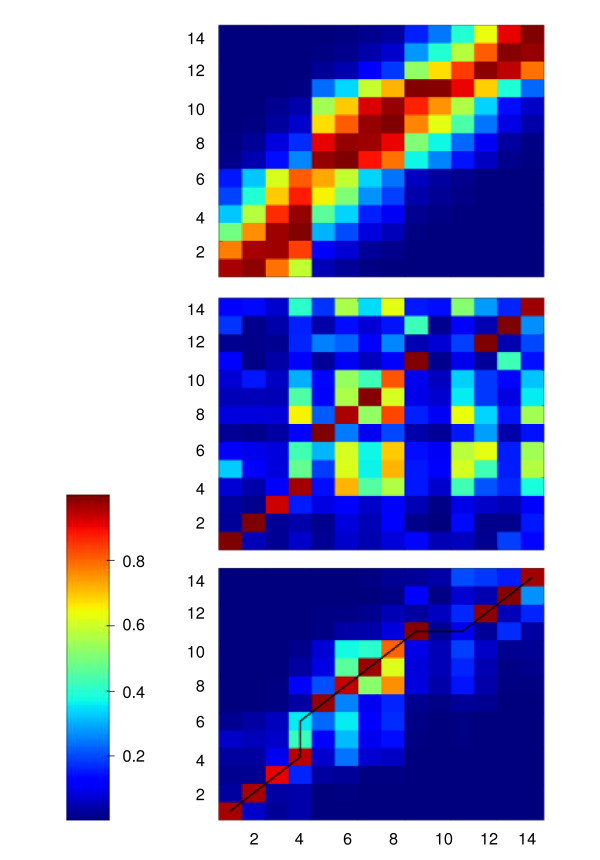
**The score matrix for dynamic programming peak alignment**. The score matrices for the alignment of two peak lists obtained from the GC-MS segments shown in Figure 3. Three matrices are shown with peak similarity (color coding is according to the scale between 0 and 1). For each score matrix the two axis refer to two peak lists to be aligned, with 14 signal peaks in each list (stationary phase peaks plotted along the x-axis and mid-log phase peaks plotted on the y-axis). The top panel shows the score matrix based on the retention time similarities only (as given the second term in the Equation [2]). The middle panel shows the score matrix based on the similarities in mass spectra taken at the peak apex (as given by the first term in the Equation [2]). The bottom panel is the total peak similarity function, as given by the Equation [2] (this is element-by-element product of the retention time and mass spectra score matrices). The traceback resulting from the application of dynamic programming is also shown in bottom panel. From the traceback the best alignment of peaks was deduced, as shown in Figure 3(b).

An example pairwise alignment is shown in Figure 3. Figure 3(a) shows TIC segments between 467 s and 534 s for two GC-MS profiling experiments of *L. mexicana *promastigotes harvested in logarithmic and stationary growth phases, respectively. Peak finding has resulted in fourteen peaks in each segment as shown in Figure 3(b), which also shows the peak matching after the alignment. Figure 4 shows different score matrices and the traceback used to deduce the optimal alignment.

### Alignment of two alignments

Consider an alignment between two internally fixed alignments: one consisting of N peak lists (henceforth referred to as N-alignment) and the other consisting of M peak lists (M-alignment). In order to find the best alignment between two such alignments with two-dimensional dynamic programming the scoring scheme must be extended.

Let the N-alignment contain K peak positions and the M-alignment contain L peak positions. In this case the score matrix may have rows indexed by the peak positions of the N-alignment, and columns indexed by the peak positions of the M-alignment, resulting in a two dimensional table with K rows and L columns. During the dynamic programming procedure the cell (i,j) of this table is filled with the score W(i,j) calculated for the i-th position of the N-alignment and the j-th position of the M-alignment, possibly modified by the gap penalty G. Alternative definitions of W(i,j) are possible. In our test implementation W(i,j) was calculated as the average similarity between the peaks in the i-th position of the M-alignment and j-th position of the N-alignment. For example, if the position i of the N-alignment contained peaks p_i1_, p_i2_,... p_iN _and the position j or the M-alignment contained peaks q_j1_, q_j2_,... q_jM _then:

W(i,j)=∑a=1N∑b=1MP(pia,qjb)∑a=1N∑b=1MI[P(pia,qjb)>0]
 MathType@MTEF@5@5@+=feaafiart1ev1aaatCvAUfKttLearuWrP9MDH5MBPbIqV92AaeXatLxBI9gBaebbnrfifHhDYfgasaacH8akY=wiFfYdH8Gipec8Eeeu0xXdbba9frFj0=OqFfea0dXdd9vqai=hGuQ8kuc9pgc9s8qqaq=dirpe0xb9q8qiLsFr0=vr0=vr0dc8meaabaqaciaacaGaaeqabaqabeGadaaakeaacqWGxbWvcqGGOaakcqWGPbqAcqGGSaalcqWGQbGAcqGGPaqkcqGH9aqpdaWcaaqaamaaqahabaWaaabCaeaacqWGqbaucqGGOaakcqWGWbaCdaWgaaWcbaGaemyAaKMaemyyaegabeaakiabcYcaSiabdghaXnaaBaaaleaacqWGQbGAcqWGIbGyaeqaaOGaeiykaKcaleaacqWGIbGycqGH9aqpcqaIXaqmaeaacqWGnbqta0GaeyyeIuoaaSqaaiabdggaHjabg2da9iabigdaXaqaaiabd6eaobqdcqGHris5aaGcbaWaaabCaeaadaaeWbqaaiabdMeajjabcUfaBjabdcfaqjabcIcaOiabdchaWnaaBaaaleaacqWGPbqAcqWGHbqyaeqaaOGaeiilaWIaemyCae3aaSbaaSqaaiabdQgaQjabdkgaIbqabaGccqGGPaqkcqGH+aGpcqaIWaamcqGGDbqxaSqaaiabdkgaIjabg2da9iabigdaXaqaaiabd2eanbqdcqGHris5aaWcbaGaemyyaeMaeyypa0JaeGymaedabaGaemOta4eaniabggHiLdaaaaaa@6D14@

where I is the indicator function and P is given by Equation [2]. Here we extend the definition of the peak similarity function to include scoring of a peak with a gap, which is always zero (i.e. P(i,-) = P(-,j) = 0). Therefore, it is possible that the denominator is less than NM, since some of the terms in Equation [3] may be zero due to involvement of gaps.

Consider the simple case, an alignment between the alignment L_A_:L_B _given by the Equation [1] and a single peak list *L*_*C *_= [r1,r2,...,rnC
 MathType@MTEF@5@5@+=feaafiart1ev1aqatCvAUfKttLearuWrP9MDH5MBPbIqV92AaeXatLxBI9gBaebbnrfifHhDYfgasaacH8akY=wiFfYdH8Gipec8Eeeu0xXdbba9frFj0=OqFfea0dXdd9vqai=hGuQ8kuc9pgc9s8qqaq=dirpe0xb9q8qiLsFr0=vr0=vr0dc8meaabaqaciaacaGaaeqabaqabeGadaaakeaacqWGYbGCdaWgaaWcbaGaeGymaedabeaakiabcYcaSiabdkhaYnaaBaaaleaacqaIYaGmaeqaaOGaeiilaWIaeiOla4IaeiOla4IaeiOla4IaeiilaWIaemOCai3aaSbaaSqaaiabd6gaUnaaBaaameaacqWGdbWqaeqaaaWcbeaaaaa@3B66@]. We note that the alignment L_A_:L_B _is a 2-alignment, and the peak list L_C _could be viewed as 1-alignment. According to Equation [3], the similarity function between the first position in the alignment given by Equation [1] (position (p_1_, q_1_)) and the first peak from the list L_C _(peak r_1_) will be calculated as:

W((p1,q1),r1)=12(P(p1,r1)+P(q1,r1))
 MathType@MTEF@5@5@+=feaafiart1ev1aaatCvAUfKttLearuWrP9MDH5MBPbIqV92AaeXatLxBI9gBaebbnrfifHhDYfgasaacH8akY=wiFfYdH8Gipec8Eeeu0xXdbba9frFj0=OqFfea0dXdd9vqai=hGuQ8kuc9pgc9s8qqaq=dirpe0xb9q8qiLsFr0=vr0=vr0dc8meaabaqaciaacaGaaeqabaqabeGadaaakeaacqWGxbWvcqGGOaakcqGGOaakcqWGWbaCdaWgaaWcbaGaeGymaedabeaakiabcYcaSiabdghaXnaaBaaaleaacqaIXaqmaeqaaOGaeiykaKIaeiilaWIaemOCai3aaSbaaSqaaiabigdaXaqabaGccqGGPaqkcqGH9aqpdaWcaaqaaiabigdaXaqaaiabikdaYaaadaqadaqaaiabdcfaqjabcIcaOiabdchaWnaaBaaaleaacqaIXaqmaeqaaOGaeiilaWIaemOCai3aaSbaaSqaaiabigdaXaqabaGccqGGPaqkcqGHRaWkcqWGqbaucqGGOaakcqWGXbqCdaWgaaWcbaGaeGymaedabeaakiabcYcaSiabdkhaYnaaBaaaleaacqaIXaqmaeqaaOGaeiykaKcacaGLOaGaayzkaaaaaa@51D9@

The similarity function between the third position of the alignment L_A_:L_B _and the first peak from the list L_C _is W(r_1_, (p_1_, -)) = P(r_1_,p_1_) since P(r_1_,-) = 0. In the case of N = 1 and M = 1, the alignment reduces to a simple pairwise alignment discussed above.

It is useful to devise a measure of how good one alignment is relative to another comparable alignment. Given an alignment between an N-alignment and M-alignment we calculate a total alignment score T as follows:

T=∑kZk
 MathType@MTEF@5@5@+=feaafiart1ev1aaatCvAUfKttLearuWrP9MDH5MBPbIqV92AaeXatLxBI9gBaebbnrfifHhDYfgasaacH8akY=wiFfYdH8Gipec8Eeeu0xXdbba9frFj0=OqFfea0dXdd9vqai=hGuQ8kuc9pgc9s8qqaq=dirpe0xb9q8qiLsFr0=vr0=vr0dc8meaabaqaciaacaGaaeqabaqabeGadaaakeaacqWGubavcqGH9aqpdaaeqbqaaiabdQfaAnaaBaaaleaacqWGRbWAaeqaaaqaaiabdUgaRbqab0GaeyyeIuoaaaa@3522@

where Z_k _is the alignment score for the position k in the alignment, and the summation is over all positions in the alignment. The value of Z_k _depends on whether the gap was inserted in the k-th position of the alignment or not. If the gap was inserted either in the N- or M-alignment then Z_k _= -G. If the gap was not inserted then Z_k _equals W(i,j) given by the Equation [3], where i is the position from the N-alignment and j is the position from the M-alignment that are aligned to one another to result in the k position of the final (N+M)-alignment.

### Alignment of an arbitrary number of peak lists

Consider the alignment of U peak lists L_A_, L_B_, L_C_,... where the peak list L_A _contains a total of n_A _peaks, the list L_B _contains a total of n_B _peaks, the list L_C _contains a total of n_C _peaks, and so on. The overall goal of the alignment process is to align the peak lists L_A_, L_B_, L_C_,... to obtain the alignment L_A_:L_B_:L_C_:... This alignment can be represented as a table or a matrix with U rows, each corresponding to one peak list. The exact number of columns in the alignment table will depend on the optimal alignment, but must be equal or greater than the maximum of n_A_, n_B_, n_C_,...

Although it is possible to devise an exact dynamic programming solution for a multi-dimensional alignment, this quickly leads to a computationally intractable problem [33,35]. Therefore we modelled our solution after the progressive multiple sequence alignment [32,33], with changes to accommodate the unique requirements of peak alignment. To find the best alignment of peak lists L_A_, L_B_, L_C_,... we first calculate all possible pairwise alignments between the peak lists. From all pairwise alignments the alignment score for each pair of peak lists is calculated (T_AB_, T_AC_, T_BC_,...). In the next step, a dendrogram (guide) tree is built which provides the similarity relationship between the peak lists. A progressive pairwise alignment is performed following the branching order given by the guide tree. In the first step the two most similar peak lists are selected and aligned, resulting in a 2-alignment. The other peak lists are added gradually following the guide tree until all peak lists are exhausted.

### Within-state and between-state alignment

In the case of experiments performed on different cell states with more than one replicate experiment per cell state it is reasonable to align first replicate experiments performed on each cell state ("within-state alignment"), and then to align the resulting alignments ("between-state alignment") [22]. This is because within each cell state one deals with true experimental replicates, and in the hypothetical case of perfect reproducibility all peaks will be observed in all experiments. In experiments performed on different cell states, such as wild-type and mutant cells, some metabolites may be missing in one state relative to another, and the expectation that all peaks observed in one state will be present in the other state is no longer valid.

Consider an alignment of three cell states (wild-type (*WT*), mutant-1 (*M1*), and mutant-2 (*M2*)), each having eight replicate experiments. In the first step, within-state alignments are performed resulting in three 8-alignments (WT, M1, and M2). To solve the order of between-state alignment all possible pairwise alignments between WT, M1, and M2 alignments are created, the total alignment scores are calculated, and another guide tree is built. The between-state alignment is built progressively from WT, M1, and M2 alignments following the guide tree to result in the final 24-alignment WT:M1:M2.

In practice, the reproducibility in peak retention times of experiments performed on the same cell state is often better compared to experiments performed on different cell states. Therefore, for optimal alignment involving different cell states it may be useful to use different parameters for retention time tolerance (D) and gap penalty (G). We denote these parameters D_w_, G_w _for within-state alignment and D_b_, G_b _for between-state alignment.

### Testing

Metabolite profiling studies were performed on different cultivated stages of the human parasite, *Leishmania mexicana*. Comparisons were also made between wild type parasites and a mutant cell line lacking three major glucose transporters [36]. Polar metabolites were analyzed by GC-MS and peak lists generated manually or by using the proposed alignment method to assess the accuracy of the alignment and the sensitivity of the latter approach to input parameters such as the gap penalty and the retention time tolerance.

### Within-state alignment of the wild-type cells profiling experiments

Eight replicate extracts of *L.mexicana *wild-type cells were analyzed by GC-MS and peaks were manually pre-processed and aligned. In this way a correct peak alignment table was constructed from 1,337 signal peaks from all eight replicate experiments. The manual analysis showed 173 unique metabolites, and the final alignment table contained 1,384 fields arranged in 8 columns (replicate experiments) and 173 rows (metabolites).

**Figure 5:**
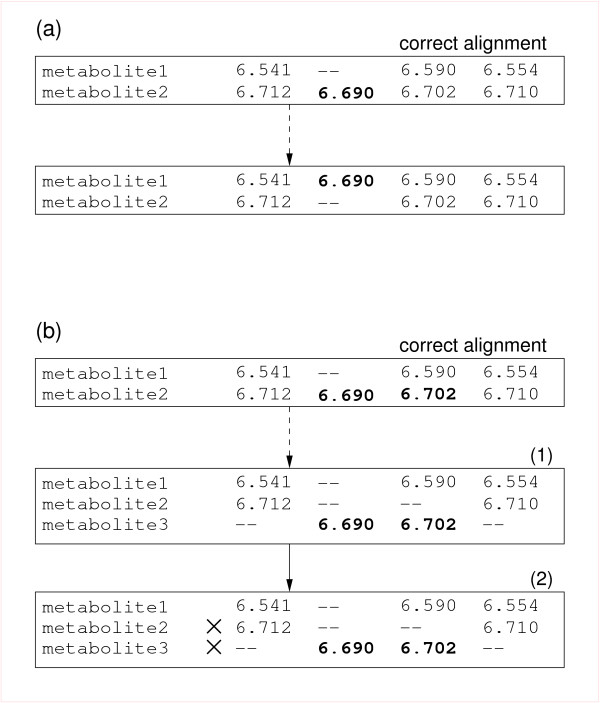
**Errors in peak alignment**. A portion of the hypothetical alignment table with two types of errors that occur in within-state peak alignment highlighted. The rows of the table represent metabolites and column represent individual experiments. The numbers shown are peak retention times in minutes. Panel (a) shows the type A error (peak mixing) where one or more peaks are shifted to an incorrect metabolite row. Panel (b) shows the type B error (metabolite splitting) where the metabolite row is split to create an artificial metabolite in the alignment table. The condition of minimum peaks is often imposed in practice (see Methods), in which case this type of error results in the deletion of the artificial metabolite (nevertheless the original metabolite is affected as it contains one or more missing peaks). If in the example shown on panel (b) it is assumed that the minimum peak cut-off is three peaks, and therefore both metabolite1 and metabolite2 will be deleted from the alignment table as the net result of the splitting error and the removal of spurious metabolite rows.

To assess the accuracy of the proposed approach a series of alignments was constructed by applying dynamic programming alignment with different input parameters. The resulting alignment tables were compared to the correct alignment table and analyzed for errors. Two types of errors were observed (Figure 5): peak mixing (type A error) and metabolite splitting (type B error). In peak mixing errors one or more peaks are shifted to a different metabolite row to take a position of a missing peak. Figure 5(a) shows the simplest case of one metabolite being shifted from metabolite-1 to a metabolite-2 row. In practice mixing can involve more than two metabolite rows. In metabolite splitting, one or more peaks are moved to create an extra metabolite row (i.e. metabolite 3 in Figure 5(b)). As a consequence of the removal of spurious metabolites (see Methods) most artificially created metabolite rows due to metabolite splitting will be discarded. The only exception would be metabolites with all eight peaks present, subject to the splitting that has resulted in two metabolite rows with exactly four peaks per row.

Comparison of the obtained peak alignment tables with the ideal alignment table allowed counting of errors. In the case of a simple peak mixing error (such as shown in Figure 5(a)) two metabolites are affected, resulting in an error count of two. If three metabolites were affected by peak mixing three errors were counted. In the case of a metabolite splitting error one or more metabolites may be affected.

**Figure 6:**
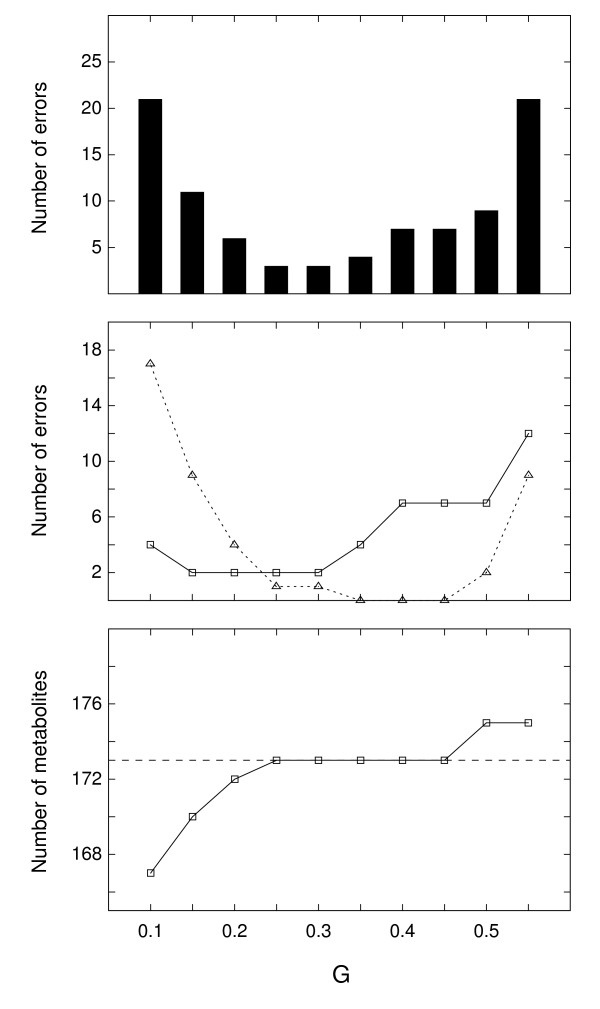
**The accuracy of peak alignment as a function of gap penalty**. The accuracy of dynamic programming peak alignment as a function of gap penalty G, shown on the x-axis. Eight replicate experiments of *L mexicana *polar extracts were processed, generating eight peak lists. The peak lists were aligned with the dynamic programming for a range of the gap penalty parameter between 0.10 and 0.55 (the retention time tolerance was fixed D = 2.5 s), and the resulting alignment tables were compared to the correct alignment table built manually. The top panel shows the total number of errors in the alignment. The middle panel shows the number of errors of type A (solid line) and type B (dashed line). The bottom panel shows the total number of metabolites in the resulting alignment. The correct number of metabolites is 173, shown in the dashed line.

Figure 6 shows the number of errors in a peak alignment table as a function of gap penalty in the range G = 0.10 to 0.55, for the fixed retention time tolerance of D = 2.5 s. For small values of G, metabolite splitting errors (type B) predominated, while for large G values peak mixing errors (type A) predominated. The decrease in the total number of metabolites for low values of G (Figure 6, bottom panel) is counterintuitive, since low values of G would favor the insertion of gaps, which in turn would create more metabolite rows in the alignment table. This effect was however more than countered by the removal of spurious metabolites rows in post-processing (see Methods). In addition, some cases of metabolite splitting resulted in a complete removal of the metabolite from the alignment table, due to the effect shown in Figure 5(b).

**Figure 7:**
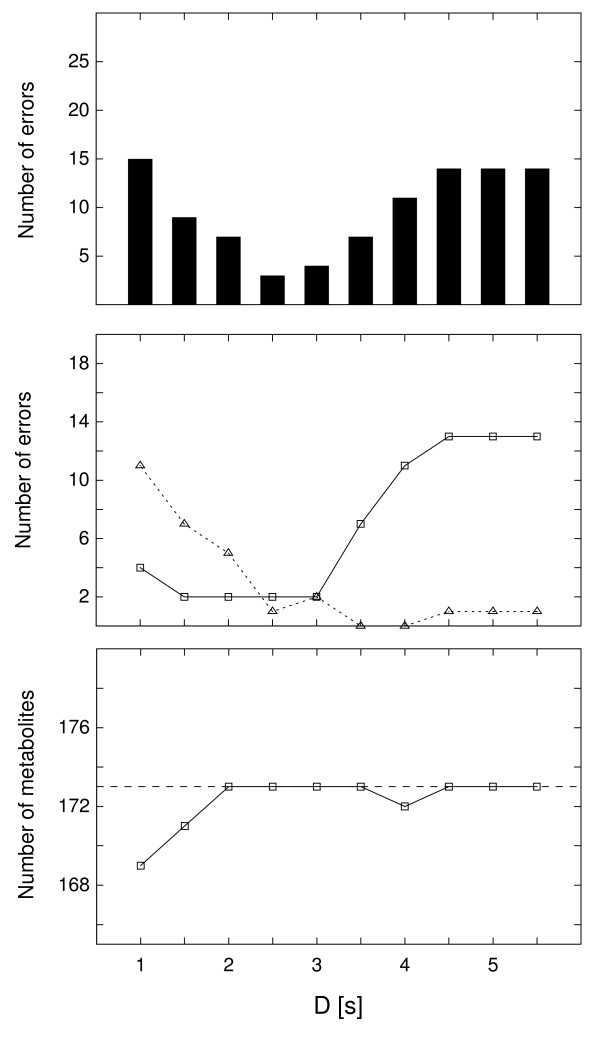
**The accuracy of peak alignment as a function of retention time tolerance**. The accuracy of dynamic programming peak alignment as a function of retention time tolerance D, shown on the x-axis. Eight replicate analyses of *L. mexicana *polar extracts were processed, resulting in eight peak lists. The peak lists were aligned with the dynamic programming for a range of retention time tolerances between 1.0 s and 5.5 s (the gap penalty was fixed G = 0.30), and the resulting alignment tables were compared to the correct alignment table built manually. The top panel shows the total number of errors in the alignment. The middle panel shows the number of errors of type A (solid line) and type B (dashed line) as explained in the text. The bottom panel shows the total number of metabolites in the resulting alignment. The correct number of metabolites is 173, shown in the dashed line.

A similar picture was observed for a number of errors as a function of the retention time tolerance (Figure 7). Small values of D favor metabolite splitting. This is because for small D any subset of peaks with small but systematically different retention times (due to experimental drift for example) would appear as a different metabolite and would be moved into a different metabolite row. However this effect was countered by the removal of spurious metabolites, and therefore a decrease in the total number of metabolites was observed for low D. Large values of D diminished peak discrimination by retention times resulting in an increase in mixing errors (type A), especially for the parts of the chromatogram which contained compounds with similar mass spectra.

### Alignment of replicate analyses of wild type and mutant metabolite extracts

These analyses were extended to replicate GC-MS chromatograms collected on wild-type and mutant cell states. The input for the alignment consisted of peak lists from 16 experiments (8 independent wild-type and 8 independent mutant extracts; Table 1) with a total of 2,665 signal peaks. Within-state alignment was performed with D_w _= 2.5 s and G_w _= 0.30, and resulted in 173 unique metabolites for the wild-type cells and 171 unique metabolites for the mutant cells. For the between-states alignment we used the same gap penalty as for within-state alignment (G_b _= 0.30), while the retention time penalty was set to D_b _= 10.0 s, roughly the value that would allow matching of peaks in the case of large retention time shifts shown in Figure 2. In practice the retention time tolerance can be estimated from the largest retention time shifts between experiments (for data shown in Figure 2 this is 5–7 s). For peaks 10 s apart the peak similarity will equal 0.6 times the similarity in their mass spectra, if D = 10.0 s (Equation [2]).

**Table 1:** Experiments used in peak alignment

**Cell state**	**Replicates**	**Aver. No peaks**	**Metabolites**
wild-type, stationary phase	8	167.1 ± 2.3	173
Δgt, stationary phase	8	166.0 ± 4.9	171

A summary of parameters obtained for replicate analyses (8 each) of polar extracts from wild type and mutant (Δgt) parasites, processed using the peak alignment method. Shown is the number of experimental replicates for each cell state, the average number of peak per replicate experiment, and the number of unique metabolites after the within-state peak alignment with D_w _= 2.5 s and G_w _= 0.30.

The complete alignment of wild-type and glucose transporter mutant experiments resulted in a total of 188 unique metabolites. The correct alignment table involving all 16 experiments was also compiled manually. A total of three errors were found in the within-state alignment of the wild-type experiments, and two errors were found in the within-state alignment of the mutant experiments. A comparison with the correct alignment table showed that incorrect between-state alignment affected a total of ten metabolites. This involved a segment of five misaligned metabolite rows between 10.2 and 10.5 minutes that had a prominent but anomalous feature at m/z = 155 that had created an effect of artificially similar mass spectra. It would be possible to remedy errors of this type with a more stringent retention time tolerance.

## Discussion and Conclusion

GC-MS is a robust and sensitive platform for the profiling of certain metabolite classes [1,4,12,13,15]. Due to experimental limitations inherent in all chromatographic separations, slight drifts are observed in GC-MS elution times between experiments [17,18]. These drifts are particularly problematic for non-targeted metabolic profiling studies, which aim to analyze all detectable analytes within multiple experiments [1,12,13,19,22,23]. There are two schools of thought regarding how to address the retention time correction in hyphenated mass spectrometry (Figure 1). Algorithms that rely on time domain data only (Figure 1, branches 1 and 3) ignore highly pertinent information contained in the m/z dimension. Accumulated evidence suggests that effective retention time correction cannot be achieved based on retention time only, regardless of whether the approach involves peak matching or profile alignment [17-19,22,26,28,29].

Profile alignment approaches that use the full chromatogram data matrices are expected to be the most accurate (Figure 1, branch 4). These approaches, however, come at a high cost, both in terms of complexity and computational costs. For example, Bylund and co-authors used correlation optimised warping [26], while Baran and co-authors used dynamic time warping with explicitly specified time shifts [30]. In both cases, an arbitrarily chosen "target" chromatogram was used to align all other chromatograms in a pairwise fashion, with the chromatogram data matrices segmented to facilitate the alignment [27,30]. This raises several difficulties. For example, it is unclear how the choice of the target chromatogram may affect the final alignment. In principle, a more objective alignment of chromatogram data matrices could be achieved by calculating a similarity tree [32,33]. This, however, must involve all possible pairwise alignments, which is likely to be computationally expensive. Furthermore, in the reported examples the chromatogram segmenting was based on strategic or node positions influenced by user chosen parameters [27], or a "representative" set of peaks [30]. This in turn raises the question of how the segmenting method might affect the final alignment. Finally, these approaches handle a large amount of uninformative noise data, since only a small portion of the full chromatogram data matrix is informative signal.

Peak matching algorithms that use both time domain data and mass spectra are particularly promising, and have been applied to both LC-MS [20,21] and more recently to GC-MS data [23]. The input for these algorithms are signal peaks extracted from full chromatogram data matrices. Since the majority of uninformative data is discarded in the peak detection step, these methods operate on a vastly reduced data set while retaining highly selective information contained in the mass spectra. Here we propose an approach that falls into this category, and uses signal peak "objects" which are signals extracted from the chromatogram data matrix. A peak object may be characterized with several attributes including peak retention time (the time taken at the peak apex), peak mass spectrum (the m/z vector taken at the peak apex), experiment/cell state information, a unique peak ID, and so on. The result of an experiment is a list of peak objects, henceforth referred to as a peak list. From this viewpoint the peak matching problem is reduced to the alignment of peaks between multiple peak lists. If we assume that the elution order of peaks is conserved (a reasonable assumption for GC-MS; also an assumption widely used in proteomics based LC-MS [37]), this problem shows resemblance to extensively studied problem of multiple sequence alignment [32,33]. In order to cope with rapidly escalating computational costs of an exact, multidimensional dynamic programming solution, efficient algorithms were developed for multiple sequence alignment [33,35]. We have adapted this approach to the problem of peak alignment in multiple GC-MS experiments.

The method proposed here uses both peak retention times and mass spectra, and relies on dynamic programming to find the optimal solution to the global alignment problem. Any peak matching method that uses both similarity in retention times and mass spectra similarity depends on a balance between the two, and devising an approach that balances this correctly poses a considerable challenge. For example, it is possible to incorporate mass spectra similarity into the total peak similarity score used in progressive hierarchical clustering [22], but it is unclear how to weight the relative contributions of retention time and mass spectra. The problem here is that progressive clustering relies on a single cutoff of the dendrogram tree to delineate peak clusters (essentially, the cutoff is a constant across the entire data set), while the retention times drifts are highly non-linear (Figure 2 and [21]). In the work of Styczynski et al, the peak matching approach used to find conserved metabolites in GC-MS metabolic profiling experiments incorporated both the similarities in mass spectra and peak retention times [23]. However, in the demonstration of this approach the retention time similarity was taken into account only coarsely, with an elution similarity threshold of 1 min [23]. When two metabolites have distinct mass spectra, the retention time information can be neglected altogether and correct peak matching can still be achieved. However, for metabolites that elute in close proximity to one another, and give similar fragmentation patterns, the information provided by retention times is critical for a correct matching. Therefore, we would expect the method of Styczynski and co-authors to have difficulty when metabolites with similar fragmentation patterns elute in close proximity, as demonstrated by their inability to resolve isoleucine and leucine in the test data set [23].

We propose the peak similarity function that incorporates the similarity between peak mass spectra modulated by the similarity in peak retention times (Equation [2]). This function is governed by two parameters: the gap penalty function (G) and the retention time tolerance (D). The first parameter (G) determines how similar mass spectra must be for peaks to be considered to represent the same metabolite; the second parameter (D) is related to expected drifts in retention times between experiments. For example, if retention times in a particular set of experiments are highly reproducible, decreasing the retention time tolerance will enable this information to be leveraged for increased accuracy in the peak alignment.

To achieve the alignment of multiple peak lists, we rely on progressive alignment based on a similarity tree. The similarity tree is calculated from pairwise alignments, and the global alignment is built progressively, starting from the two most similar peak lists, and joining other peak lists in a process guided by the similarity tree [32,33]. When several cell states with multiple replicate experiments per state are analyzed, within each cell state one deals with true experimental replicates, while in experiments performed on different cell states some metabolites may be missing altogether in one state relative to another. To accommodate for this complexity, we first perform a within-state alignment, followed by the between-state alignment built by aligning the within-state alignments. In the case of more than two sets of replicate experiments, a similarity tree is built based on pairwise similarities between fixed within-state alignments, and then the alignments themselves are aligned progressively following the similarity tree. Therefore multiple guide trees are built, one for each set of replicate experiments to facilitate within-state alignment. An additional guide tree may be built to facilitate between-state alignment if more than two states are present.

Several methods for peak matching based on both retention times and mass spectra (Figure 1, branch 2) have been described recently [20,21,23]. Of these only the approach of Styczynski and co-authors was developed on GC-MS data [23], while the software packages MZmine [20] and XCMS [21] were developed on LC-MS data. In our experience the latter software packages tend to overinterpret GC-MS data, assigning a greater number of peaks than expected (probably related to differences in fragmentation patterns between GC-MS and LC-MS). Nevertheless, the alignment algorithms implemented in MZmine and XCMS are relevant and we briefly review them here.

MZmine uses a simple alignment method to build the peak alignment table: one peak at a time is taken and an attempt is made to match it to an existing row of the peak alignment table. If no rows match a new row is created [20]. A secondary peak detection method is used to fill the gaps in the resulting alignment table [20]. We expect this approach to exhibit limitations similar to those observed in hierarchical clustering [22], and discussed above.

XCMS incorporates one of the most advanced peak matching algorithms for metabolite profiling data described to date [21]. In this approach the distribution of peaks along the time domain by using the kernel density estimator is calculated, and regions where many peaks have similar retention times are identified [21]. From this, a fixed time interval that determines each group of peaks is deduced [21]. In practice, the retention time drifts are distributed in a highly irregular fashion, and a fixed time interval is unlikely to be able to capture peak groups correctly. This is suggested by observed peak collisions, where more than one peak from the same experiment is joined into a single group [19]. The same problem was encountered in other peak matching methods [19,22], and requires additional, empirical intervention, such as "collision resolution" [19] or "tie-breaking" in XCMS [21]. The approach we propose inherently prevents peak collisions. Furthermore, it does not rely on any fixed intervals to group peaks, nor does it rely on any assumptions about the distribution of peaks across the samples.

It is of interest to compare the method proposed here to the approach for constructing signal maps, described recently in LC-MS proteomics experiments [37]. Underlying the calculation of signal maps is the method for optimal alignment of chromatogram data matrices, that can be classified as belonging to branch 4 in Figure 1. These authors used the Needleman-Wunsch algorithm to align full chromatogram data matrices obtained from LC-MS proteomics experiments [37]. They also used minimum spanning tree, or progressive merging of pairwise alignments into a consensus run, to produce a global signal map [37]. Apart from the overt difference in that we focus on extracted signals rather than using the full chromatogram data matrices, there are several important yet more subtle differences between the two methods. First, in contrast to the method of [37] the score function proposed here incorporates both similarities in retention times and mass spectra. Second, in constructing signal maps from peptide mass spectra Prakash and co-workers allow for multiple mass spectra in one experiment to correspond to a single mass spectrum in the other experiment [37]. Since we are aligning peak objects rather than the raw signal, our approach inherently allows only one-to-one peak matching, and we consider explicitly the question of gaps (a match-to-nothing). Both of these features are critical for the ability to achieve the alignment of signal peaks in GC-MS. Similarly as in the approach of [37], we rely on the two-dimensional formulation of the global sequence alignment problem. However, during progressive alignment of individual experiments we do not merge individual runs into a "consensus" run, because this has a potential to degrade signal if two unrelated signal peaks are merged. Rather, our approach is based on the generalized solution for the alignment of two alignments, i.e. an N- and M-alignment. This has an additional benefit to be directly extensible to the alignment of pre-computed alignments representing replicates of different cell states (where signal-to-nothing matches may be significant), and allows one to tackle the problem of an arbitrary number of cell states with the same conceptual framework and finite computational resources. We note that the modification proposed for the calculation of mass spectra similarity [37], and results from other proteomics studies [38], could be used in the method proposed here.

It would be useful to compare more directly the performance and accuracy of the approach proposed here to the approaches for peak matching described previously. Unfortunately, this is currently not feasible for two reasons. Firstly, a standard data set which could be used as a benchmark does not exist. Furthermore, it is difficult to create such a data set *ad hoc*. Most other alignment methods of interest, such as methods implemented in software packages MZmine [20] or XCMS [21], are embedded in the multi-step processing pipelines, and the input to peak matching is generated directly from the output of package-specific peak detection. Secondly, a suitable metric to quantify differences between two methods does not exist. This is particularly problematic, because in even the simplest case the resulting alignment table may involve hundreds of peak entries, and it is not clear how to represent and quantify the differences between two such tables.

The development of a suitable metric to compare two peak matching methods is likely to require a separate research effort. To circumvent this we have performed a detailed analysis of absolute error and compiled error statistics relative to the "correct" alignment table created manually (Figures 6 and 7). The error analysis performed on experimental data consisting of eight replicate GC-MS experiments collected on *Leishmania *parasites with ~170 peaks per experiment showed that, for near optimal parameters, <5 metabolites were affected by misalignment errors. This approaches the accuracy achieved in a manual alignment, which required tens of man-hours for the tested data set.

The alignment accuracy was not overly sensitive to empirical parameters for a wide range of values (gap penalty and retention time tolerance), suggesting that the method is robust. To address the problem of a benchmark data set, we provide our test data set in the supplementary material (see Additional files 1, 2, 3). This includes input peak lists, raw chromatogram data matrices, and alignment tables, including those produced for optimal parameters, as well as the "correct" alignment table prepared manually.

The main drawback of the proposed approach is that it operates on a set of signal peaks obtained from peak detection pre-processing. Automated peak detection remains a challenge [30], and any errors introduced during peak detection (such as missing peaks or false peaks) will propagate through to the alignment tables. This is inherent in all peak matching methods, and could be viewed as an advantage as well. Focusing on signal peaks results in a greatly reduced data set which contains the vast majority of interesting signal, and allows one to leverage this information for downstream processing [21], in addition to significantly reducing computational costs required for downstream processing.

## Methods

### Metabolic profiling of *L.mexicana*

*L. mexicana *wild-type and *L. mexicana *Δgt promastigotes (lacking three glucose transporters [36] were cultivated in RPMI media, pH 7.4 containing 10 % fetal calf serum (FCS). Parasites were harvested in mid-log phase or stationary phase (24 hr and 96 hr after inoculation, respectively). Prior to harvesting, the culture medium was rapidly chilled to 0°C by immersing the culture flasks in a dry-ice/ethanol bath. The temperature of the medium was monitored with an electronic thermometer and subsequent steps performed at 0°C. Replicate aliquots of the quenched culture medium (containing 4 × 10^7 ^cells) were harvested by centrifugation (15,000 g, 30 sec, 0°C) and washed twice with ice-cold phosphate-buffered saline, pH 7.5. The washed cell pellets were suspended in chloroform:methanol:water (containing 1 nmole *scyllo*-inositol and 1 nmole norleucine internal standards) (1:3:1 v/v/v) and extracted at 60°C for 15 min. Water was added to give a final chloroform:methanol:water ratio of 1:3:3 (v/v) and the aqueous and organic phases separated by centrifugation. The upper aqueous phases were dried *in vacuo*, suspended in 20 mg/ml methoxyamine in pyridine (20 μl, 16 hr, 25°C) with continuous shaking and then derivatized with MSTFA + 1% TMCS (Pierce; 20 μl, 1 hr, 25°C). Samples (1 μL) were injected onto an Agilent 6890N gas chromatograph interfaced with a 5973 mass selective detector using a 7683 automatic liquid sampler. Gas chromatography was performed on a 30 m DB5-MS column with 0.25 mm inner diameter and 0.25 μm film thickness (J&W Scientific). Injection temperature was 270°C, the interface set at 250°C, and the ion source adjusted to 230°C. The carrier gas was helium (flow rate 1 ml/min). The temperature program was 1 min isothermal heating at 70°C, followed by a 12.5°C/min oven temperature ramp to 295°C, then 25°C/min to 320°C and held for 1 min. Mass spectra were recorded at 3.2 scans/s (m/z 50–500).

### Post-processing and data preparation

The total ion-chromatogram was integrated in ChemStation (MSD Chemstation D.01.02.16, Agilent Technologies) by using the default integrator. Resulting peak tables were exported to external files for further processing, together with raw data in ANDI-MS format. Initially the TICs were examined visually for each experiment, and peak lists were edited manually to mark the reference peak and uninformative peaks originating from the derivatizing reagent (TMS). Subsequently the reference peak and uninformative peaks were removed from the peak lists, and peaks in the region 6.5 to 21.0 min of each chromatogram were selected for further processing. Prior to peak alignment the mass spectra at peak apexes were extracted from the ANDI-MS files, and signals at m/z 73 and 147 (largely due to the derivatizing reagent) were suppressed for all peaks. To remove spurious metabolites such as those that arise as artefacts of empirical peak integration, all metabolite rows with less than four peaks were discarded after the alignment (i.e. one half of the original sample size of eight replicate experiments) [22].

## Authors' contributions

MDR was involved in the development of the method and development of the test implementation. DPDS was involved in the development of GC-MS experiments and error analysis. WWK was involved in the development of the test implementation, ECS was involved in GC-MS experiments, MJM was involved in the development of the method and GC-MS experiments, TPS was involved in the development of the method. VAL originally conceived the project and was leading development of the method, development of the test implementation, and error analysis. VAL drafted the manuscript; all authors contributed to the final version. All authors have read and approved the final manuscript.

## Supplementary Material

Additional file 1 Raw data files, peak lists, and alignment tables for eight replicate experiments obtained by GC-MS metabolic profiling of wild type *L.mexicana*. This folder contains peak lists for eight GC-MS replicate experiments, raw GC-MS data files in the NetCDF format, manually prepared peak alignment table, and the alignment table obtained by automated peak alignment. Click here for file

Additional file 2 Additional raw data files for replicate experiments obtained by GC-MS metabolic profiling of wild type *L.mexicana*. This folder contains three additional raw GC-MS data files in the NetCDF format, that correspond to peak lists provided in SupplMaterial1/ChemStation. Click here for file

Additional file 3 Additional raw data files for replicate experiments obtained by GC-MS metabolic profiling of wild type *L.mexicana*. This folder contains three additional raw GC-MS data files in the NetCDF format, that correspond to peak lists provided in SupplMaterial1/ChemStation. Click here for file
